# Predictors of failed reduction after prosthetic hip dislocation

**DOI:** 10.1007/s00590-025-04589-7

**Published:** 2025-11-12

**Authors:** Nicholas Frappa, Danil Chernov, Aidan G. Papalia, Samuel Fuller, Ellen Lutnick, Matthew G. Alben

**Affiliations:** 1 Jacobs School Of Medicine and Biomedical Sciences, University at Buffalo, State University of New York, Buffalo, USA; 2 Department of Orthopaedic Surgery, Geisinger Health System, Danville, USA; 3 Department of Orthopaedics and Sports Medicine, University at Buffalo, State University of New York, Buffalo, USA

**Keywords:** Prosthetic hip dislocation, Closed reduction, Procedural sedation, Emergency department, Anesthesia, Total hip arthroplasty

## Abstract

**Purpose:**

Prosthetic hip dislocation remains one of the most common complications following total hip arthroplasty (THA), often managed with closed reduction under procedural sedation. This study aimed to evaluate how sedation strategy, procedural location, and patient-specific factors influence the success of closed reduction in prosthetic hip dislocations. We hypothesized that higher body mass index (BMI), use of dissociative agents, and emergency department (ED) setting would be associated with lower reduction success.

**Methods:**

A retrospective review was performed of adults presenting with prosthetic hip dislocations to two tertiary centers (2014–2024). Demographic, procedural, and pharmacologic data were abstracted from medical records. Logistic regression was used to assess associations between reduction success and location, sedation class, BMI, and procedural duration. Receiver operating characteristic (ROC) analysis identified thresholds for escalation.

**Results:**

Among 201 reduction encounters (111 patients), overall success was 73.1% (ED 54.3%, OR 94.4%). In multivariable analysis, non-ED location was the dominant predictor of success (OR 38.4, 95% CI 8.0–184; *p* < 0.001), while no medication class independently predicted outcome. Procedure duration was inversely associated with success in the ED (OR 0.88 per minute; *p* = 0.047), with an escalation threshold of approximately 25 min (AUC 0.72). BMI, age, comorbidities, and implant type were not associated with reduction success.

**Conclusion:**

This study demonstrated reduction location was the dominant predictor of success, with non-ED settings demonstrating substantially higher odds of achieving closed reduction. Longer procedure duration was inversely associated with success, suggesting that protracted or repeated attempts should prompt early operative escalation. Standardized sedation protocols, adequate muscle relaxation, and algorithmic triage for high-risk cases may enhance success rates and reduce resource utilization in the management of prosthetic hip dislocations.

## Introduction

Prosthetic hip dislocations are one of the most common complications following total hip arthroplasty (THA), affecting approximately 0.3% to 10% of primary THAs and up to 25% of revision cases [1–3]. It remains a leading cause of hospital readmission and revision surgery, with significant implications for healthcare utilization, patient satisfaction, and functional recovery [1–3]. Recurrent instability is also common: more than half of patients re-dislocate after an initially successful reduction, and rates may approach 80% after hemiarthroplasty dislocation [4, 5]. Prompt reduction is essential to prevent neurovascular compromise, alleviate pain, and restore joint stability.

Initial management of prosthetic hip dislocations typically occurs in the emergency department (ED), where closed reduction under procedural sedation is frequently attempted [6, 7]. This approach is favored for its efficiency, cost-effectiveness, and the potential to avoid closed versus open reduction in the operating room (OR). However, failure to achieve reduction in the ED often necessitates operative intervention increasing patient morbidity, length of stay, and healthcare costs [7]. Although numerous reduction techniques and sedation agents are available, there remains no consensus regarding optimal pharmacologic strategies or criteria for selecting patients who are most likely to benefit from bedside versus reduction in the operating room.

Pharmacologic agents commonly used for ED sedation include propofol, ketamine, midazolam, etomidate, and opioid analgesics, often in combination [8–10]. Propofol provides rapid-onset sedation but lacks analgesia and can cause dose-dependent hypotension and respiratory depression [9]. Ketamine offers dissociative sedation with preserved airway reflexes and potent analgesia, making it appealing in emergency settings [9]. However, its sympathomimetic effects and potential for emergence phenomena can complicate its use [9]. Comparative evidence supports this complementary use, with studies showing that ketamine–propofol combinations reduce respiratory adverse events compared with propofol alone and that both agents provide safe and effective procedural sedation in the ED with only minor differences in recovery characteristics and no difference in procedural success [11, 12]. In addition, the time course over which the agents are administered also plays a role.

Beyond pharmacologic choice, patient-specific factors such as obesity, dislocation direction, and comorbidities may influence the likelihood of successful reduction. Elevated body mass index (BMI) has been associated with increased risk of recurrent dislocation and poorer outcomes after conservative management of THA dislocation, likely reflecting both mechanical and soft-tissue factors that complicate reduction and stability [13]. However, few studies have examined whether BMI directly impacts the immediate success of closed reduction, which remains poorly characterized despite its clinical importance.

While prior studies have evaluated pharmacologic agents used for procedural sedation in the emergency department, few have specifically investigated how sedation strategy and patient-specific factors influence the success of prosthetic hip dislocation reductions This study aimed to evaluate the relationship between sedation regimen, patient characteristics, and procedural outcomes in patients undergoing reduction for prosthetic hip dislocations. We hypothesized that higher BMI and use of adjunctive dissociative agents would be associated with failed ED reduction, and that ED-based reductions would be less successful than those performed in the OR or post-anesthesia care unit (PACU).

## Methods

### Ethics

Internal Institutional Review Board Approval was granted for this study. All subjects provided informed consent prior to enrollment.

## Study design

We conducted a retrospective review of adult patients (age ≥ 18 years) who presented with prosthetic hip dislocations to two affiliated tertiary care centers between January 1, 2014, and July 20, 2024. Patients were identified using institutional electronic medical records by searching diagnosis codes related to hip dislocation (specifically, CPT 27266) between the dates of January 1, 2014 and July 20, 2024 Data was abstracted from the medical record independently by two medical researchers.

Inclusion criteria were adult patients (≥ 18 years) with a diagnosis of prosthetic hip dislocation who underwent a documented reduction attempt. Patients were excluded if they had incomplete medical records, absence of documented sedation details, or lack of a recorded reduction attempt.

## Hip reduction procedure

Reductions attempted in the ED are typically attempted by an orthopedic resident with supervised conscious sedation via emergency department staff. Medication used for sedation is at the discretion of the emergency department attending physician, including consideration for provider level of comfort with certain medications, and patient comorbidities/risk factors.

Reductions in the operating room or post-anesthesia care unit (PACU) are typically attempted by orthopedic attending/resident, supervised by orthopedic attending, and sedation supervised by anesthesiologist. In PACU, conscious sedation is utilized, and medications are picked at the digression of the attending anesthesiologist supervising the procedure; in the operating room the patient is typically fully sedated, including intubation, and medications promoting muscle relaxation are utilized.

### Statistical analysis

Collected variables included patient demographics (age, sex), BMI, comorbidities (hypertension, diabetes, hyperlipidemia, coronary artery disease, COPD, dementia, substance use), dislocation direction, reduction location (ED, OR, PACU), sedation regimen, procedure duration, time from dislocation to reduction, and reduction outcome (success vs. failure). IIn ED analyses, propofol alone was defined as propofol use without adjunct agents, whereas propofol + any indicated propofol used with one or more additional medications. When sample sizes permitted, specific two-agent combinations (e.g., propofol + ketamine) were also summarized. Medications were grouped by pharmacologic class for regression models: anesthetic (propofol, etomidate), dissociative (ketamine), opioid (fentanyl), paralytic (rocuronium, succinylcholine), and sedative (midazolam). Counts were reported by individual agents for descriptive summaries and by drug class for regression analyses.

Analyses were performed primarily at the encounter level, treating each closed reduction attempt as a unique observation (201 total encounters). A dislocation episode was defined as a distinct dislocation event for a patient, which could include multiple reduction encounters (157 total episodes). Patient characteristics (111 unique patients) were summarized once per patient using the earliest episode, and episode-level analyses (index attempt only) were conducted as sensitivity checks. Age was calculated from date of birth to dislocation (or reduction when unavailable), and dislocation-to-reduction intervals were expressed in days. Comorbidities were binarized and summed into a composite count. Analyses used available cases without imputation; denominators are reported in the Results.

Descriptive statistics were calculated for all variables (mean ± SD and/or median [IQR] for continuous variables; counts and percentages for categorical variables). Comparisons between groups used Wilcoxon rank-sum tests for continuous variables, Kruskal–Wallis for ≥ 3 groups, and Fisher’s exact tests for categorical variables (χ² where appropriate). For direction-specific age comparisons, pairwise tests were adjusted using the Benjamini–Hochberg method. Where informative, effects sizes (e.g., Cliff’s delta and Vargha–Delaney A) are reported with 95% confidence intervals.

To estimate adjusted associations with successful reduction, logistic regression models were fitted. The all-locations model included reduction location (ED vs. non-ED), age, sex, BMI, procedure duration, dissociative use, and—in a separate specification—the five medication classes. An ED-only model included age, sex, BMI, procedure duration, and dissociative use. Odds ratios (ORs) with 95% confidence intervals are presented. All tests were two-sided, with *p* < 0.05 considered statistically significant. Receiver operating characteristic (ROC) analysis was performed to assess the discriminatory ability of procedure duration for predicting reduction failure in the ED subgroup. Optimal and high-specificity thresholds were identified using the Youden index and specificity-based coordinates.

Analyses were performed in R 4.5.0 using tidyverse, dplyr, ggplot2, pROC, lubridate, broom, tableone, janitor, effsize, gt, flextable, and officer.

## Results

### Baseline characteristics

We identified 201 reduction encounters, representing 157 dislocation episodes in 111 patients. Baseline characteristics are summarized at the patient level, with outcomes and regression analyses conducted at the encounter level unless otherwise specified. Of 109 patients with sex recorded, 76 (69.7%) were female. Median age was 69 years (IQR 60–77; *n* = 110) and median BMI 27 kg/m² (IQR 23–34; *n* = 61).

Comorbidities were classified at the patient level if documented in any encounter. The most common were hypertension (60%) and hyperlipidemia (36%), followed by COPD (16%), diabetes (9%), coronary artery disease (8%), and dementia (13%). Substance use (alcohol or drug) was documented in 10% of patients, and tobacco use in 15%. Full patient-level characteristics are provided in Table 1.

At the encounter level, age, BMI, and composite comorbidity count did not differ between successful and unsuccessful reductions (all *p* > 0.5).

**Table 1:** Patient-level baseline characteristics

Characteristic	Value
Patients, n	111^a^
Sex recorded, n	109
Female, n (%)	76/109 (69.7%)
Male, n (%)	33/109 (30.3%)
Age, years (mean ± SD)	68.8 ± 12.9
Median [IQR]	69.4 [60.0–76.7]
BMI, kg/m² (mean ± SD)	28.6 ± 7.3
Median [IQR]	27.3 [23.4–34.1]
Comorbidities, n (%)	
Hypertension	66/111 (59.5%)
Hyperlipidemia	40/111 (36.0%)
COPD	18/111 (16.2%)
Diabetes mellitus	10/111 (9.0%)
Coronary artery disease	9/111 (8.1%)
Dementia	14/111 (12.6%)
Substance use (alcohol or drug)	11/111 (9.9%)
Tobacco use	17/111 (15.3%)
Implant type recorded, n	105
Total hip arthroplasty, n (%)	95/105 (90.5%)
Hemiarthroplasty, n (%)	10/105 (9.5%)
^a^IQR = interquartile range.	

## Reduction location and outcomes

Reduction outcome was documented for 197/201 encounters; 144/197 (73.1%) achieved successful closed reduction. Success varied by location: ED 57/105 (54.3%), OR 85/90 (94.4%), PACU 2/2 (100%). In a multivariable model including age, sex, BMI, procedure duration, dissociative use, and location, non-ED location remained strongly associated with higher odds of success (OR 38.4, 95% CI 8.00–184; *p* < 0.001) (Fig. 1).

## Procedure duration

**Fig. 1:**
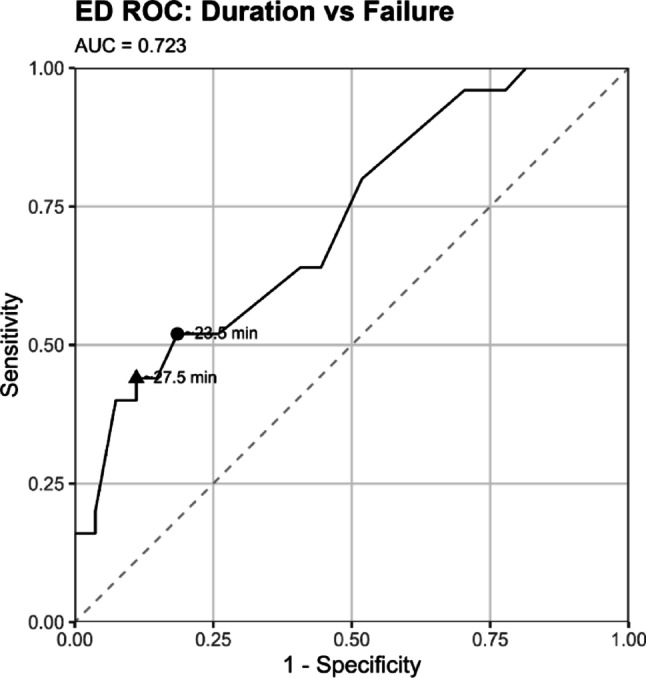
Receiver operating characteristic (ROC) curve for procedure duration predicting reduction failure in emergency department (ED) encounters

### Dislocation direction

Dislocation direction was recorded in 172/201 encounters: posterior 70/172 (40.7%), superior 42/172 (24.4%), anterior 39/172 (22.7%), and lateral 21/172 (12.2%). The dislocation-to-reduction interval was available in 87/201 encounters (median 0 days [0–1]; mean 9.1 ± 78.4 days; right-skewed).

In the ED, success did not differ by direction (posterior 19/37 (51.4%), anterior 6/19 (31.6%), lateral 6/11 (54.5%), superior 17/25 (68.0%); *p* = 0.127). Across the full cohort, age differed by direction (*p* = 0.033); pairwise testing showed anterior dislocations occurred in younger patients than posterior (median 63 vs. 70 years; *p* = 0.038), with effect sizes Cliff’s Δ = 0.32 (95% CI 0.10–0.50) and Vargha–Delaney A = 0.66 (95% CI 0.55–0.75).

### Agents used in reduction

Medications were documented in 177/201 encounters (88.1%). Among these, anesthetics (propofol or etomidate) were used in 168/177 (94.9%), dissociatives (ketamine) in 24/177 (13.6%), opioids (fentanyl) in 46/177 (26.0%), paralytics (rocuronium or succinylcholine) in 53/177 (29.9%), and sedatives (midazolam) in 10/177 (5.6%). Medication use by location is summarized in Table 2A and B.

**Table 2:** (A) operating room (OR) medication use (B) emergency department (ED) medication use

Medication	Used (*n*/*N*, %)
(A)	
Propofol	73/90 (81.1%)
Ketamine	5/90 (5.6%)
Midazolam	32/90 (35.6%)
Fentanyl	46/90 (51.1%)
Rocuronium	30/90 (33.3%)
Succinylcholine	53/90 (58.9%)
Etonamide	8/90 (8.9%)
(B)	
Propofol	93/107 (86.9%)
Ketamine	24/107 (22.4%)
Midazolam	3/107 (2.8%)
Fentanyl	1/107 (0.9%)
Rocuronium	0/107 (0.0%)
Succinylcholine	1/107 (0.9%)
Etonamide	6/107 (5.6%)

Within the ED subgroup, dissociative use was not associated with successful reduction: 41.7% success with ketamine vs. 58.0% without (*p* = 0.171; OR 0.52, 95% CI 0.18–1.43). When examining propofol-based regimens specifically, reductions performed with propofol alone achieved 58.5% success (38/65), compared to 42.9% success with propofol in combination with one or more adjunct agents. This difference did not reach statistical significance (*p* = 0.182). Single add-on groups, reported descriptively due to small sample sizes, included propofol + ketamine with 8/20 (40.0%) successes (OR 2.09, 95% CI 0.68–6.78; *p* = 0.201). All other propofol combinations occurred in two or fewer encounters.

In the multivariable model across all locations, which included anesthetic, dissociative, opioid, paralytic, and sedative classes along with age, sex, BMI, procedure duration, and location, no medication class was independently associated with successful reduction (all *p* > 0.20). Location remained the dominant predictor, with markedly higher odds of success outside the ED.

### Implant type

Implant type was ascertainable in 194/201 encounters; 181/194 (93.3%) were total hip arthroplasties (THA) and 13/194 (6.7%) were hemiarthroplasties. Patients with hemiarthroplasty were older than those with THA, 75 vs. 66 years (*p* = 0.001) and had a lower BMI, 26.4 vs. 30.2 kg/m^2^ (*p* = 0.014). Overall closed-reduction success was similar by implant type (THA 132/180 (73.3%) vs. hemiarthroplasty 9/12 (75.0%); *p* = 1.00). In the ED subgroup, success was 55/99 (55.6%) for THA and 1/4 (25.0%) for hemiarthroplasty (*p* = 0.329), though estimates were imprecise given the small hemiarthroplasty sample. In multivariable logistic regression across all locations (adjusting for age, sex, BMI, procedure duration, dissociative use, and location), implant type was not independently associated with success (THA vs. hemiarthroplasty *p* = 0.25), whereas non-ED location remained strongly associated with higher odds of success (OR = 87.9, 95% CI 13.2–584; *p* < 0.001).

### Emergency department subgroup

Among ED reductions with outcome recorded (*n* = 105), 57 (54.3%) were successful. Procedure duration was inversely associated with success (OR 0.883 per minute; 95% CI 0.780–0.998; *p* = 0.047). Age and BMI were not associated with successful reduction in the ED (both *p* > 0.15). Medication and implant findings for the ED subgroup are summarized above.

## Discussion

This retrospective study identified procedural location as the principal determinant of successful closed reduction in prosthetic hip dislocations, with patient- and medication-related variables exerting a lesser influence once adjusted for confounders. While reductions performed in the operating room or post-anesthesia care unit were almost uniformly successful, those attempted in the emergency department achieved successful reduction in only 54% of encounters. Multivariable modelling confirmed that non-ED reductions had markedly higher odds of success, emphasizing the importance of the procedural environment, anesthesia support, and depth of muscle relaxation in determining outcome. These findings suggest that patient triage and procedural planning should prioritize settings equipped for general anesthesia when risk factors for reduction difficulty are present.

After accounting for procedural and patient variables, no pharmacologic class, including dissociative agents such as ketamine, was independently associated with successful reduction. Kruggel et al. [10] evaluated methohexital (anesthetic), ketamine, and propofol for 43 musculoskeletal reductions and found comparable rates of success and respiratory adverse events across agents. Similarly, a meta-analysis by Yan et al. [12] compared ketamine–propofol combination with propofol alone and found no difference in overall adverse events, though respiratory complications were slightly fewer in the combination group. Miner et al. [11] also reported that both propofol and ketamine provided safe and effective procedural sedation in the ED, with similar procedural success but longer recovery times and more frequent emergence agitation observed with ketamine. In addition, Willman and Andolfatto [14] demonstrated that ketamine–propofol combination sedation (“ketofol”) was safe and effective for ED procedural sedation, supporting its complementary pharmacologic profile in this setting. A more recent meta-analysis by Sharif et al. [15] found that propofol was associated with shorter recovery times compared with midazolam–opioid combinations, whereas ketamine–propofol combinations produced higher patient satisfaction but increased neurological adverse events. Collectively, these findings are consistent with broader procedural sedation literature showing that the choice of sedative agent exerts limited influence on procedural success.

Ketamine is frequently utilized in ED procedural sedation due to its potent analgesic properties and favorable hemodynamic profile. However, unlike agents such as propofol and benzodiazepines, ketamine does not induce deep skeletal muscle relaxation; rather, it maintains or even increases muscle tone [9, 14]. Ketamine–propofol combination sedation (“ketofol”) has demonstrated effectiveness and favorable recovery profiles in pediatric orthopedic reductions [16, 17]; however, these findings may not be directly translatable to adult prosthetic hip dislocations. Within the ED subgroup, dissociative use demonstrated a trend toward lower success but did not reach statistical significance, suggesting that these agents may be used more often in challenging cases rather than directly impairing the likelihood of reduction. While ketamine’s preservation of skeletal muscle tone could theoretically hinder manipulation of the prosthetic joint where complete relaxation is advantageous, this relationship was not observed in the present study, and other procedural factors warrant further investigation. Prospective studies with standardized dosing and neuromuscular blockade protocols would help clarify whether medication regimen or depth of sedation is the primary driver of procedural success.

Notably, and as expected, the pattern of medication use differed sharply between operating room and emergency department settings. OR reductions were almost uniformly performed under general anesthesia, with frequent use of neuromuscular blockade (succinylcholine in 59% and rocuronium in 33%) and adjunctive agents such as midazolam and opioids. In contrast, ED reductions relied almost exclusively on propofol-based procedural sedation, with minimal or no paralytic use; this is consistent with the fact that neuromuscular blockers require airway control and are not part of standard ED sedation protocols [18]. This pharmacologic disparity, inherent to the differing environments and levels of anesthesia support, likely explains much of the observed difference in reduction success. Complete neuromuscular relaxation achievable under general anesthesia facilitates controlled manipulation of the prosthetic joint and reduces the periarticular resistance that often limits closed reduction in awake or semi-sedated patients [19]. Conversely, procedural sedation in the ED preserves varying degrees of skeletal muscle tone, even with dissociative or deep sedative agents, which can impede reduction. Thus, while medication class itself was not independently predictive in multivariable analysis, the physiologic conditions achievable under general anesthesia—rather than specific drug choice—appear central to the markedly higher success rate observed outside the ED.

Procedure duration was inversely associated with successful reduction, particularly within the ED setting, where longer manipulations carried lower odds of success (OR 0.88, *p* = 0.047). However, this relationship is expected rather than causal: once a hip is reduced, the procedure ends, whereas unsuccessful or technically complex reductions continue until escalation occurs. Duration therefore reflects procedural difficulty and the persistence of challenging cases rather than serving as a determinant of failure. ROC analysis (AUC 0.72) identified an escalation threshold of approximately 25 min (the midpoint between the Youden-optimal cutoff [23.5 min] and the high-specificity point [27.5 min]) beyond which continued bedside attempts were rarely successful. Early transition to the operating room, where anesthesia depth and muscle relaxation can be optimized, may help achieve the markedly higher success rates observed outside the ED.

Neither implant type nor patient factors such as age, BMI, or comorbidity burden demonstrated a significant association with reduction success. Although previous studies have identified high BMI as a contributor to dislocation risk and technical difficulty [13, 20], our results suggest that such patient characteristics may be outweighed by modifiable procedural conditions. The lack of association between implant type and reduction outcome indicates that management principles are broadly applicable across total and hemiarthroplasty dislocations. Additionally, prior biomechanical and clinical studies have shown that the effects of morbid obesity on hip stability arise largely from soft-tissue and positioning mechanics rather than intrinsic patient or implant characteristics [20, 21].

These findings complement prior work demonstrating that the quality of sedation and procedural environment are critical to achieving favorable outcomes. Dela Cruz et al. [8] reported fewer complications and faster recovery with propofol-based sedation compared with etomidate or opioid/benzodiazepine combinations, while Waseem et al. [22] found high success rates when reductions were performed under standardized propofol protocols in the ED. The lower ED success rate observed in the present cohort parallels the 62% success reported by Frymann et al. [23] for reductions performed under conscious sedation in the emergency department, suggesting that real-world heterogeneity in sedation depth, provider experience, and muscle relaxation likely contributes to variability in outcomes. Integrating structured decision pathways and early anesthesia involvement may improve both procedurasl efficiency and patient safety.

### Limitations

This study has several limitations. Its retrospective design restricts causal inference and relies on accurate documentation of medications, timing, and duration. Sedation depth, specific drug doses, and the experience level of the operator were not recorded and may have introduced confounding. Incomplete or inconsistent documentation of procedural sedation parameters is common in emergency settings, as noted in prior audits of sedation records [24]. Implementation of a structured quality-improvement program—such as standardized sedation checklists, electronic documentation templates, and feedback on completion rates—could enhance data reliability and patient safety in future studies. The precise interval between the index arthroplasty and subsequent dislocation was unavailable for most patients, preventing assessment of early versus late instability patterns. Similarly, implant-specific variables such as femoral head size, surgical approach, and component positioning could not be extracted from the medical record but may influence reduction difficulty and recurrence risk. Detailed radiographic data describing implant wear, mobilization, or periprosthetic fracture were also unavailable for many encounters, limiting evaluation of implant condition and its contribution to reduction success. The single-institution design may limit generalizability, and small subgroup counts constrained model precision for certain agents and implant types. Finally, because procedure duration was abstracted from chart documentation rather than continuous monitoring, brief pauses or undocumented manipulations may have affected recorded times. Despite these limitations, the consistent and robust association between procedural location and reduction success across multiple analytic models supports the validity of our findings.

## Conclusion

This study demonstrated reduction location was the dominant predictor of success, with non-ED settings demonstrating substantially higher odds of achieving closed reduction. Longer procedure duration was inversely associated with success, suggesting that protracted or repeated attempts should prompt early operative escalation. Standardized sedation protocols, adequate muscle relaxation, and algorithmic triage for high-risk cases may enhance success rates and reduce resource utilization in the management of prosthetic hip dislocations.

## Data Availability

No datasets were generated or analysed during the current study.
